# “We Don’t Have Access to the Village”: Barriers and Facilitators to Optimal Parenting Experiences Among Deaf Parents

**DOI:** 10.3390/ejihpe16070106

**Published:** 2026-07-22

**Authors:** Julie C. Mitchiner, Tahleen A. Lattimer, Gigi Zheng, Emma Kane, Gabrielle Majeri, Christina Whetsel, Jessica Contreras, Michael M. McKee, Peter C. Hauser

**Affiliations:** 1 Department of Education, Gallaudet University, Washington, DC 20002, USA; 2 Department of Physical Medicine & Rehabilitation, University of Michigan, Ann Arbor, MI 48104, USA; tahleenw@med.umich.edu; 3 NTID Research Center on Culture and Language, Rochester Institute of Technology, Rochester, NY 14623, USAgm9760@g.rit.edu (G.M.); pchgss@rit.edu (P.C.H.); 4 Department of Family Medicine, University of Michigan Medical School, Ann Arbor, MI 48104, USAmmmckee@med.umich.edu (M.M.M.); 5 Department of Leadership, Research, and Policy, University of Colorado, Colorado Springs, CO 80918, USA; jcontr11@uccs.edu

**Keywords:** Deaf, parenting services, parent–child relations, family structure, Deaf culture, qualitative research

## Abstract

Deaf parents experience higher rates of healthcare barriers and adverse parenting outcomes compared with their hearing peers. This qualitative study explored the parenting experiences of Deaf parents to better understand barriers and facilitators to optimal parenting situations. Semi-structured, individual remote interviews were conducted with twenty Deaf parents in the United States who primarily communicate using American Sign Language (ASL) and have children aged 13 years old and under. Interviews examined experiences related to access to information and resources, relationships with healthcare providers, communication accessibility, and involvement with the educational system. Data were analyzed using thematic analysis. Four major themes emerged: communication accessibility across parenting contexts, lack of accessible parenting-related information and services, family dynamics, and the role of assistive technologies in parenting. Participants reported that systemic barriers across healthcare, educational, and service systems create persistent communication challenges and delays, disempowering experiences, and contribute to a critical lack of resources designed for Deaf parents. These findings underscore the need for increased awareness and improved communication access for Deaf parents across healthcare, educational, and legal settings to support positive parenting experiences.

## 1. Introduction

Deaf people comprise a marginalized and stigmatized group who may have experienced challenges in both informal and formal learning related to parenting. From a medical perspective, being Deaf is a medical condition that is a problem with an individual’s body. However, under the socioecological perspective, disability is a societal problem where there are barriers and attitudes towards different types of bodies (Mauldin, 2020; Mitra, 2006; Shakespeare, 2010) wherein Deaf individuals experience deaf-related stigma or negative biases known as audism (Aldalur & Pick, 2023; Holcomb, 2010; Tomaszewski et al., 2025). The Minority Stress Theory (Meyer, 2003), which has been supported with empirical evidence from different non-deaf marginalized and stigmatized groups (see Hoy-Ellis (2023) for review), postulates that these individuals experience barriers, oppression, and discrimination that can cause excessive stress. In this study, we explored the parenting experiences of Deaf parents to better understand barriers and facilitators to optimal parenting situations.

Deaf parents are more likely, than their hearing peers, to experience risk factors for their own wellbeing and that of their children. For example, Deaf parents are disproportionately likely to lack social support, experience interpersonal violence (Anderson & Kobek Pezzarossi, 2014; Anderson & Leigh, 2011; Ballan et al., 2017; Fellinger et al., 2012; Pollard et al., 2014), experience marital discord, be involved in child custody disputes (Abedi et al., 2018), have involvement with child protective services and struggle with inaccessible legal and court systems (Powell & Albert, 2021). Deaf parents, due to their marginalization, miss incidental learning opportunities, and thus are disproportionately likely to have significant literacy, health literacy and knowledge gaps (McKee et al., 2026; Pollard & Barnett, 2009). Similarly, to other disadvantaged populations who struggle with inadequate health literacy, children of parents with inadequate health literacy have poorer health and developmental outcomes (Griese et al., 2020; Scotten, 2015). This is believed to be largely due to low navigational ability or literacy (Griese et al., 2020; Scotten, 2015). Additionally, Deaf parent’s children more often experience poor educational progress, compared to children of hearing parents (Singleton & Tittle, 2000). At the same time, systemic factors in the health, education, and legal sectors render service providers ill-prepared to support Deaf parents. Within the legal system, accommodations may not be provided to parents in a timely manner, parents may struggle to secure attorneys due to communication needs and / or lack of accommodations, and during custody disputes judges may exhibit biases in favor of the hearing partner or spouse (Wilks, 2025). In the educational realm, parents often report that accommodations are not provided during meetings with their children’s teachers and other school personnel (St. Clair et al., 2025; Kanwal et al., 2022), including at meetings to develop children’s Individualized Education Programs (IEPs), as required under the Individuals with Disabilities Education Act (National Association of the Deaf, n.d.; Trahan, 2016) In the health care setting, there are concerns about health care provider stigma (Helm et al., 2023; Ratakonda et al., 2025), inaccessible communication (St. Clair et al., 2025; James et al., 2022), and health literacy (McKee et al., 2026), and knowledge gaps including childhood developmental milestones, immunizations and children care.

While there have been a few studies on these issues as they manifest within the healthcare and child protection systems (i.e., Powell & Albert, 2021), the data pertaining to most of these issues is largely anecdotal, based on observations from various legal, medical, social and educational providers. Deaf parents often struggle in navigating through these different parenting-based resources, strategies and requirements, placing themselves at increased risk for adverse parenting outcomes (McKee et al., 2026). Given the limited research on the parenting experiences of Deaf parents, this exploratory qualitative study sought to better understand how Deaf parents describe their parenting experiences, including the barriers and facilitators that shape optimal parenting situations. The study was guided by the following research questions:

1. How do Deaf parents describe their experiences navigating parenting in family, community, educational, and service contexts?
2. What barriers do Deaf parents identify as adversely affecting their ability to have optimal parenting situations?
3. What facilitators, supports, or resources do Deaf parents perceive as helpful in promoting positive parenting experiences?

## 2. Materials and Methods

### 2.1. Theoretical Framework and Researcher Positionality

This study was guided by a phenomenological qualitative approach, which seeks to understand and interpret the lived experiences of individuals from their own perspectives while acknowledging the influence of researchers’ positionality in the research process (see Alhazmi & Kaufmann, 2022, for discussion). The analysis was further informed by a socioecological perspective, recognizing that parenting experiences are shaped at multiple levels, including individual, interpersonal, organizational, community, and societal contexts. This framework allowed us to examine how Deaf parents’ experiences are influenced not only by personal and family factors but also by healthcare systems, educational institutions, policies, and broader societal attitudes.

Researcher positionality was intentionally considered throughout the study. Deaf community members and researchers were involved in all stages of the research. All but one of the authors are Deaf ASL signers, two are Hispanic, two are Asian, and five are parents themselves. The first author is a Deaf mother and an educator with expertise in early childhood and Deaf Education research. Another author is a Deaf father and a primary care physician with clinical and research expertise in Deaf health and provides care for patients of all ages, including children. Another author is a Deaf mother who serves as the head of a school for the deaf, and another is a Deaf father, psychologist, and director of a research center focused on Deaf individuals’ cultural and language experiences. Collectively, the research team’s lived experiences and professional expertise enhanced cultural and linguistic responsiveness while fostering reflexivity throughout the research process. At the same time, the team engaged in ongoing discussion to examine assumptions, challenge interpretations, and ensure that findings remained in participants’ narratives rather than researchers’ personal experiences.

### 2.2. Design

This qualitative study used a phenomenological design to examine the parenting experiences of Deaf parents, with the goal of gaining a deeper understanding of their successes, as well as the barriers and facilitators that influence parenting outcomes. The outcomes focus on parents’ ability to navigate their parenting experiences within medical, educational, and legal contexts. A qualitative approach was appropriate for this study, as it centers on participants’ lived experiences of raising children and identifying challenges, strengths, and resource gaps. Data were collected through semi-structured, remote interviews conducted across the United States. Interviews with 20 Deaf parents were conducted, a sample size chosen based on data saturation. Participants were asked about their access to parenting information and resources, communication access, satisfaction with healthcare providers, teachers, and other child-related professionals, and experiences with the healthcare, educational, and legal system throughout their child-rearing period. Deaf community members and researchers were involved in all stages of the research.

### 2.3. Participants

This study focused on the subset of Deaf parents who preferred to communicate in American Sign Language (ASL) as their primary language. To be eligible for participation, Deaf parents had to be 18 years of age or older and have at least one child aged 13 years or younger residing in the United States. Children aged 13 years and younger were the focus of this study because parents are typically more actively involved in healthcare, educational, and developmental decision-making during early childhood and early adolescence than during later adolescence (Dotterer, 2022). Participants were recruited by a mix of purposive, convenience, and snowball sampling via email, social media, and in-person recruitment at community events. To maximize diverse representation, study flyers and social media graphics were distributed widely, targeting organizations serving Deaf people, including within different ethnic communities. A total of 20 Deaf parents participated in the study, ranging in age from 26 to 50 years old, with a mean age of 37.5 years old. The sample represented a range of genders, racial and ethnic backgrounds, relationship statuses, and educational experience (see Table 1). Additionally, participants hailed from four major U.S. Census regions, with the largest share coming from the West (*n* = 8, 42.1%). The Northeast was also well represented (*n* = 6, 31.6%) while smaller proportions came from the South (*n* = 3, 15.8%) and Midwest (*n* = 2, 10.5%). One declined to report their state of residence but confirmed that they resided in the United States.

### 2.4. Data Collection Procedures

Ethical approval for this study was obtained from one of the authors’ Institutional Review Boards. The research team conducted 60 min-long semi-structured qualitative interviews in ASL with 20 Deaf parents. Data saturation was determined when our team recognized repeated themes of barriers, facilitators and experiences highlighted in earlier interviews. Recruitment and enrollment were stopped at that time. Interviews were conducted via Zoom video conferencing app. Prospective participants first completed a web-based questionnaire (via a Qualtrics survey) to determine their eligibility. Eligible participants were directed to a web-based informed consent, then a demographic questionnaire. Study staff contacted eligible participants to schedule interviews. After completing the interviews, participants received a $20 incentive. Interviews were video recorded for later transcription from ASL to written English by bilingual transcriptionists.

### 2.5. Analysis Procedure

Interview transcripts and information from the background survey were uploaded to the web-based qualitative analysis application Dedoose Version (Version 10.0.59) to support data management, coding, and analysis. Interviews were thematically analyzed by team members with backgrounds and training in areas including Deaf studies, health care and medicine, education, family systems, and qualitative methods. Furthermore, eight out of the nine authors identify as Deaf, which informed the team’s attention to the cultural, linguistic, and systemic dimensions of Deaf parents’ experiences. Data were analyzed using thematic analysis, following Braun & Clarke’s six-phase approach to identifying, analyzing, and reporting patterns within qualitative data (Braun & Clarke, 2006; Clarke & Braun, 2017), consistent with prior qualitative research with Deaf individuals (Ratakonda et al., 2025). The analytic process was iterative and collaborative, moving beyond code frequency alone to consider the conceptual significance, depth, and relevance of patterns across interviews. As such, we outline this six-step process in further detail below.

First, members of the research team familiarized themselves with the data by reviewing the transcripts in full and discussing initial impressions during team meetings. Second, team members generated initial codes by identifying meaningful segments of text, including phrases, sentences, and longer narrative excerpts, related to Deaf parents’ experiences across various parenting contexts. All transcripts were reviewed by at least two team members. Coding was first conducted through a close reading of the transcripts, with the coded data then managed and further examined in Dedoose. Initial codes captured both the contexts in which parenting experiences occurred, such as health care, legal, and school settings, and the nature of those experiences, including instances of information seeking and sharing, communication access, and advocacy. This also included instances in which Deaf parents felt supported, faced barriers, and their experiences and advice for other Deaf parents as they raise their children.

Third, the team met regularly to compare codes, clarify definitions, and discuss areas of disagreement. Discrepancies were resolved through discussion and consensus, with codes being refined to better reflect the data. Fourth, the team examined coded excerpts across transcripts to identify broader patterns and relationships among codes. Dedoose was used to organize coded excerpts, compare code applications, and review patterns across the dataset. While code frequency was used as one analytic tool to help identify recurring areas of and themes, frequency was not treated as the sole basis for theme development. Rather, themes were developed and labeled by considering recurrence across participants, conceptual richness, relevance to the study aims, analytic coherence, and the extent to which codes reflected meaningful barriers and/or facilitators in Deaf parents’ experiences.

Fifth, we reviewed and refined potential themes to ensure that the coded excerpts within each theme formed a coherent pattern and that distinctions between themes were analytically clear. This process involved revisiting both individual excerpts and the full dataset to ensure that themes accurately represented participant narratives. Finally, as a last step themes were defined and named to capture the central meaning of the observed patterns. This process resulted in four overarching themes: communication (in)accessibility across parenting contexts, limited resources and support, family dynamics, and technology use. To enhance analytic rigor and trustworthiness throughout this entire process, the team engaged in collaborative coding discussions, consensus building, and maintained detailed documentation of analytic decisions.

## 3. Results

Participants described parenting experiences across healthcare, education, legal, and resource contexts. Thematic analysis identified four major themes reflecting barriers and facilitators to positive parenting experiences among Deaf parents. The themes are as follows: (1) communication (in)accessibility across parenting contexts, (2) limited resources and support, (3) family dynamics, and (4) technology use. Below, each theme is discussed in detail.

### 3.1. Communication (In)Accessibility Across Parenting Contexts

Communication accessibility was heavily cited by participants as a factor that shaped their ability to engage in parenting-related decisions, cutting across healthcare, education, legal, and community settings. One context in which this was discussed related to interpreters. Notably, experiences with interpreters did vary depending on participants’ geographic location, institutional awareness, and individual advocacy efforts. For example, participants living in areas with larger Deaf communities often described systems with more consistent interpreter provision. For example, one participant (Participant 19) shared, “*I live in [city] so I am very spoiled. [It] has the largest capacity of Deaf population. Therefore, interpreters are everywhere… I went to the ER at midnight, and the interpreter and CDI took less than 5 min to get there… So I think [city] is very spoiled but to answer your question… they always have an interpreter.*” reflecting on the increased resources in their area but also recognizing this is not the norm. In contrast, other participants described persistent gaps, including delayed interpreter requests, reliance on poorly implemented Video Remote Interpreting (VRI), and pressure to communicate through written English or through a hearing partner or other person present. These barriers were especially prevalent in healthcare settings, where participants described needing but not receiving interpreters during labor and delivery, pediatric appointments, emergency visits, and follow-up care. Even when interpreters were present, communication access was not always equitable. One participant (Participant 18) described feeling ignored by a doctor who directed communication to his wife instead, explaining, “*… the doctor knew my wife is hard of hearing, he would look at my wife and ignore me in the room… I have the interpreter there, but they talked to my wife…*” This illustrates that interpreter provision alone was not sufficient when providers failed to recognize Deaf parents as active participants and decision-makers in the encounter. In several cases, parents described having to advocate for their own access in the midst of medical events, diverting their attention from their own or their child’s care to the logistics of communication.

Communication was also heavily discussed in relation to educational settings. While some schools proactively provided interpreters and asked about accommodation needs, many participants described inconsistent support during parent–teacher conferences, Individualized Education Program (IEP) meetings, and school events. Beyond the provision of interpreters, participants identified a broader lack of professional awareness of ASL, Deaf culture, and the linguistic experiences of children of Deaf adults (CODAs). For example, one parent shared how a teacher misidentified their child’s use of ASL number conventions as a mathematical error, prompting the parent to explain that the child was drawing on a different language system rather than demonstrating a lack of understanding. Such experiences suggest that meaningful communication access in educational settings requires not only language accommodations but also cultural competence among school personnel.

Another form of communication inaccessibility involved the loss of informal or incidental information. Participants described consistently being the last to learn about school illnesses, schedule changes, teacher dynamics, or peer issues, information that hearing parents often absorbed through school communication or informal conversations with other parents. One participant (Participant 2) described learning about a school illness outbreak only because of a conversation with another parent, sharing, “*I found out their son was sick, so I asked what happened. They mentioned that many kids had gotten sick at school, which surprised me… I was able to learn more because I had access to that conversation. Otherwise, I wouldn’t have known.*” The same participant later reflected on the consequences of delayed information, explaining, “*I only learned about it two or three days later, and I really wish I had known sooner… The fact that I wasn’t informed earlier is frustrating because it became clear that the illness was spreading.*” Because this information is exchanged spontaneously and informally, it is rarely captured through formal accommodations, leaving Deaf parents to actively seek out what hearing parents may receive without effort.

To remedy these gaps, some parents described having to “create their own village” through group chats and relationships with both Deaf and hearing parents. As one participant (Participant 7) explained, “*I believe it’s crucial for deaf and hard of hearing parents to research and gather the information they want… they may find it necessary to seek additional resources and connecting with other deaf and hard of hearing parents, as well as hearing parents, can be very beneficial. If they have established relationships, they should leverage those connections.*” This illustrates how parents actively built informal communication networks to compensate for gaps in formal school communication and to remain engaged in their children’s educational and social environments.

### 3.2. Limited Resources and Support

One concern the participants raised was that the vast majority of available parenting resources were English-based and designed for hearing audiences. Participants expressed a strong and consistent preference for information created in ASL by Deaf individuals or fluent signers, rather than interpreted versions of hearing-centered materials, which were rare. This distinction was particularly important for parents with varying levels of English literacy and for topics requiring nuanced understanding, such as child development. Notably, even participants with extensive higher education, professional training, or prior experience in education, healthcare, or child development described difficulty locating accessible, relevant, and Deaf-centered parenting resources. For example, one parent (Participant 7) expressed a desire for a centralized online platform, sharing, “*I felt like if we could set a community platform online where they are accessible for everyone that can in ASL, or a person who can do transcription, and add language, I know that is a dream… Having access to that kind of information would be amazing, especially since parenting styles vary so much! I would love to be able to watch and feel like I’m not alone…*” Thus, the challenge was not simply a matter of individual knowledge or literacy but reflected broader gaps in the availability and organization of resources for Deaf parents.

The lack of accessible resources was especially salient during moments of uncertainty or crisis. One participant (Participant 20) described caring for a newly adopted child with a croup and not knowing what to do, stating, “*I remembered it was the scariest moment of my life, because they did not give you a handbook on how to be a parent. And we don’t really have access to the village.*” This quote captures how resource gaps were both informational and relational. Parents needed not only written or digital information but also access to trusted networks of people who could provide practical guidance in urgent parenting situations. When resources did not exist in accessible formats, participants described relying on informal networks or doing without, both of which placed additional strain on already limited time and energy.

Participants also drew attention to the absence of resources reflecting the diversity of Deaf families. They identified needs for information and services tailored to Black, Indigenous, and people of color (BIPOC) Deaf parents; LGBTQIA+ (Lesbian, Gay, Bisexual, Transgender, Queer, Intersex, Asexual, and more) parents; DeafBlind parents; single parents; and parents of children with co-occurring disabilities such as autism. As one parent (Participant 20) shared, “*We have a limited village of other Deaf people or people who can sign… so for me, as a gay Deaf person, most of my friends don’t have kids. It is very rare for a few people who have kids and in LGBTQ community, so I was like, “I can’t contact them….*” This reflects how the size and relevance of available support networks were shaped by intersecting identities. The participant later explained that tailored resources and opportunities for LGBTQIA+ Deaf parents to connect with one another would help reduce isolation and provide more relevant forms of support.

Participants also described using digital tools to fill information gaps. One participant (Participant 21) explained how he used Google and ChatGPT when assessing a child’s symptoms, sharing, “*I googled, ‘a bruise on a four-year old’s back and is it normal?’… I resorted to using ChatGPT, and I liked the way it summarizes things for me. While Google does not indicate which links to use, I didn’t know which one to use, so ChatGPT makes sense.*” While digital tools helped some parents quickly access information, participants also emphasized the need to interpret results cautiously and to determine whether the information was appropriate for their child’s situation.

### 3.3. Family Dynamics

Family relationships functioned as both facilitators of and barriers to positive parenting experiences, and participants’ accounts revealed how deeply intertwined parenting practices were with questions of identity, communication, and intergenerational experience. Participants described profound enjoyment in watching their children grow, communicate, and develop emotional awareness, and many defined successful parenting not through milestones or external markers but through relational qualities such as trust and open communication. Notably, several participants described making intentional departures from disciplinary or negative practices they had experienced in their own upbringing, choosing instead to prioritize listening, emotional validation, and collaborative decision-making with their children. One participant (Participant 21) described feeling proud and empowered after overcoming skepticism from their own parents about their abilities to support literacy, as their children thrived academically through an English and ASL bilingual home environment focused on reading and communication. They explained “*My wife still made sure that their children are going to be bilingual with voices turned off…her [mother-in-law] telling us what to do, we are still firm with having our voices turned off and using sign language. We still read with them at home. All four kids thrived with reading—above their grade level—all of them.*”

Family dynamics varied across participants, with parents describing experiences of co-parenting, single parenting, shared caregiving, blended family structures, and extended family involvement. These structures shaped both the supports available to parents and the barriers they encountered while navigating parenting responsibilities. For some participants, partners played an important role in sharing caregiving tasks, managing sleep routines, preparing for appointments, and supporting communication with children. In these cases, shared responsibility was described as helping reduce stress and strengthen family connection. However, co-parenting also introduced challenges when partners disagreed about language use, cochlear implants, discipline, schooling, or how to respond to children’s developmental needs. These disagreements were often tied to broader questions of identity, language access, child autonomy, and what forms of communication should be prioritized in the home.

Single parents described a distinct set of challenges related to parenting. For these participants, parenting often involved managing healthcare, education, custody, and everyday responsibilities with less support. Some single parents also described having to learn legal systems on their own, locate resources independently, and make major decisions without a co-parent or partner to share the burden. Peer support from other single Deaf parents was described as especially valuable because it offered both emotional validation and practical guidance from others who understood the specific challenges of parenting while navigating systems alone.

Beyond the immediate family, peer and community networks also shaped family life. Deaf parent groups, community organizations, social media communities, friends, and Deaf cultural events provided opportunities to exchange strategies, share resources, and reduce isolation. These networks sometimes functioned as chosen families or extended networks of support, particularly for parents who lacked supportive relatives or partners. Reflecting on this, one participant (Participant 13) shared how, “*for Deaf parents, building a strong network is very important—it creates a sense of safety,*” explaining how these connections allow parents to share information but also ask questions and learn from each other’s experiences. However, access to these networks was uneven. Participants in areas with smaller Deaf communities or limited local resources described greater difficulty finding peer support that reflected their experiences. Thus, family dynamics extended beyond the household, reflecting a broader ecology of support, exclusion, and connection.

### 3.4. Technology Use

Finally, technology use was described as a meaningful part of parenting, particularly in relation to safety monitoring, communication access, and information seeking. Many parents discussed technologies related to child safety, especially for infants and young children. Participants described using visual baby monitors, flashing or vibrating alerts, smart doorbells, cameras, and apps to maintain awareness of their home environment. These tools helped parents respond to sounds and safety cues that might otherwise be inaccessible, such as an alarm sounding. Technology also supported participants’ efforts to find parenting information, with parents describing the use of internet searches, social media, apps, and artificial intelligence tools.

Participants also used technology and visual adaptations to support everyday communication with their children and others. One participant (Participant 19) described using Live Transcribe to try to follow their daughter’s conversations with peers, but also noted its limits, explaining, “*I do use Live Transcribe on my phone…If there’s a specific topic I’m concerned about, I have to scroll through countless lines to find it. Hearing parents can easily tune in and out until they catch something interesting, but as a deaf parent, I’m left in the dark.*” This illustrates how technology could provide partial access, while still failing to fully replicate the ease of incidental listening available to hearing parents. Participants also described adapting physical environments to improve visual access. For example, the same parent (Participant 19) went on to explain, “*I upgraded to a larger rearview mirror in the car because it helps me see better when I sign and move my body, making it easier to visualize the entire environment in the van.*” The participant further explained that the mirror allowed both the parent and children to see each other signing, improving safety and communication while driving.

However, participants approached technology with caution, citing concerns about misinformation, English-only content, unreliable devices, and products not designed or tested with Deaf users in mind. Overall, technology served as an important support for Deaf parenting by increasing access, safety, and independence, but participants did not describe it as a complete solution. Rather, technology was most useful when paired with accessible systems, qualified interpreters, ASL-centered resources, and responsive professional support.

## 4. Discussion

The present work sought to explore the experiences of Deaf parents, with particular attention to the barriers and facilitators that shape these experiences. Findings provide qualitative evidence that Deaf parents face persistent, systemic barriers across multiple parenting-related contexts. Consistent with prior work, participants report challenges related to healthcare, education, and legal systems, among several others (Griese et al., 2020; McKee et al., 2026). These barriers stem from inaccessible communication, limited provider awareness, and structural inequities, emerging at critical periods such as pregnancy, early childhood, and school-based decision making, potentially increasing the stress and risk for adverse parenting outcomes (Hall et al., 2026; St. Clair et al., 2025). These findings provide additional evidence for the Minority Stress Model (Meyer, 2003) as the interviews illustrate that Deaf parents experience excessive stress navigating systems that are not designed to be accessible to all.

### 4.1. Parenting Strengths Within Systemic Barriers

Across interviews, participants challenged deficit-based assumptions, or audism biases, about Deaf parenting. Rather than describing parenting challenges as rooted in Deafness itself, participants described barriers created by systems, services, and resources that were not designed with Deaf parents in mind. This finding aligns with socio-ecological models of disability that locate barriers in environmental and systemic structures rather than within individuals (Kuenburg et al., 2016; Pollard & Barnett, 2009). Parents were actively engaged in their children’s development, safety, education, and well-being. They sought information, advocated for accommodations, built networks of support, adapted technologies, and developed communication strategies within their families. Thus, findings suggest that a large majority of parenting barriers are produced by inaccessible systems, inadequate professional training, and limited Deaf-centered resources.

This distinction is important, as many participants described experiences in which their competence as parents was questioned by family members, professionals, or institutions. Such experiences are consistent with literature documenting audist and ableist biases within child welfare (Ballan et al., 2017) and legal systems (Wilks, 2025; Zidenberg, 2023). These experiences reflect broader ableist and audist assumptions that equate hearing with parental capacity. The findings demonstrate that Deaf parents do not lack the desire, knowledge, or commitment to parent effectively. Rather, they are often required to parent in environments that withhold equal access to information, communication, and decision-making. This reframing shifts the focus from individual parent deficits to systemic responsibility.

### 4.2. Communication Access

Communication access emerged as a foundational condition shaping Deaf parents’ ability to participate fully in parenting-related contexts. This finding is consistent with prior work documenting pervasive communication barriers faced by Deaf individuals in healthcare (Hall et al., 2026; Kuenburg et al., 2016; Barnett et al., 2011) and educational settings (Singleton & Tittle, 2000). Participants’ ability to make decisions, advocate for their children, and engage with healthcare providers, educators, legal professionals, and community systems depended on whether information was communicated in accessible ways. Interpreter access, ASL communication, and captioning, were not described as optional supports, but as necessary conditions for full parental participation.

Findings extend existing work on communication barriers among Deaf populations by showing how barriers occur across multiple systems simultaneously. Participants did not describe isolated moments of inconvenience. Rather, they described repeated experiences of having to request interpreters, correct inaccessible communication practices, educate professionals, or rely on less effective communication methods. In healthcare settings, these barriers affected pregnancy, birth, emergency care, pediatric care, and follow-up appointments, consistent with documented disparities in healthcare communication for Deaf patients (McKee et al., 2022). In educational settings, they affected school meetings, IEP participation, parent-teacher communication, and informal school involvement (Trahan, 2016). In legal and service systems, communication barriers created additional obstacles to understanding rights, navigating custody concerns, and advocating for accommodations (Wilks, 2025).

A particularly important finding was the loss of incidental information. Participants described missing informal information that hearing parents may receive through casual or informal exchanges with other parents and/or teachers. This finding is consistent with longstanding literature documenting the impact of incidental learning loss among Deaf individuals (McKee et al., 2026). This type of information loss is distinct from formal accommodation failures, but it still shaped parents’ ability to remain informed, connected, and involved. It is further compounded by the participants’ described loss of a “village” to tap into for expertise. For Deaf parents, communication access must therefore be understood broadly, including both formal interpretation and everyday access to the informal information that supports parenting decisions.

### 4.3. Navigation and Needs for Resources

Participants’ information-seeking behaviors reflected resourcefulness and determination but also revealed systemic gaps in the availability of accessible parenting materials. Prior research has documented that Deaf individuals often experience health literacy and knowledge gaps due to limited access to information in accessible formats rather than cognitive or intellectual limitations (Pollard & Barnett, 2009; McKee et al., 2026). In this study, similar patterns emerged: parents actively sought information but were hampered by the predominance of English-only, hearing-centered materials. The preference for resources created in ASL, rather than interpreted from English, reflects broader findings about the importance of linguistically and culturally appropriate health communication (Barnett et al., 2011; Kushalnagar et al., 2015). Digital resources, including social media and internet searches, offered some access, but participants noted concerns about misinformation and the lack of vetted, ASL-based content. Importantly, participants identified the need for intersectional resources, ones that reflect the diversity of the Deaf community, including BIPOC, LGBTQIA+, DeafBlind, single parents, and parents of autistic or otherwise neurodivergent children. This finding aligns with calls in the literature for health and parenting resources that address the intersecting dimensions of identity, culture, and disability (Ecklund, 2024; Wu & Grant, 2020).

### 4.4. Family, Peer, and Community Networks

Consistent with prior work, family dynamics emerged as both facilitators and barriers to positive parenting (see Abedi et al., 2018). Intergenerational differences in communication modality, educational philosophy, and attitudes toward Deaf identity created both tension and opportunities for growth within families. Single parenting presented distinct challenges, with parents describing the need to navigate care with less support. Research has shown that Deaf parents involved in custody disputes may face additional barriers, including judicial bias and inaccessible legal proceedings (Wilks, 2025). Peer support from other Deaf parents was especially valued, offering emotional validation and practical guidance. The Deaf community functioned as an important source of support, consistent with prior research documenting the protective role of community connection for Deaf individuals (Pollard & Barnett, 2009). However, access to these networks was uneven, particularly for those in geographically isolated areas.

### 4.5. Technology as Support, Not Substitute

Technology played a meaningful role in participants’ parenting lives, supporting safety monitoring, communication, and information access. However, technology was described as a supplement to, rather than a replacement for, direct communication access and systemic accommodations. Participants noted limitations including unreliable connectivity, products not designed for Deaf users, and the inability of technology alone to address systemic communication barriers. This finding is important in the context of increasing reliance on technological solutions (e.g., VRI) that may not adequately replace in-person interpretation or structural accessibility improvements (Hall et al., 2026). Additionally, there remains a lack of useful guides on what technological approaches or assistive devices that are designed for Deaf parents.

## 5. Implications

Findings hold several implications for parenting support, clinical practice, education, legal systems, and policy. First, there is a clear need for centralized, ASL-accessible resources specifically tailored for Deaf parents. These should be co-developed with Deaf parents and communities, reflecting the diversity of family structures, cultural identities, and communication practices within the Deaf community, especially as resources developed for hearing parents and subsequently translated are insufficient (Pollard & Barnett, 2009; Kushalnagar et al., 2015).

Second, professional training across healthcare, education, and legal systems must include education about Deaf culture, ASL communication, and the legal rights of Deaf parents to accommodations. Research has shown that inadequate clinician knowledge contributes directly to health disparities among Deaf populations (Hall et al., 2026; Kuenburg et al., 2016). Third, systemic policies must ensure timely provision of qualified interpreters across all parenting-related contexts, not only in formal healthcare and legal settings but also in informal educational interactions. Finally, research and intervention development should adopt intersectional frameworks that account for the diversity within the Deaf parenting population.

## 6. Limitations and Future Directions

The present work is not without limitations. First, our sample included a smaller sample of 20 Deaf parents who primarily communicated in ASL. While these interviews provided rich qualitative data, findings may not represent the full diversity of Deaf parenting experiences. A key limitation of this parenting project is that most participants identified as female (65%), non-Hispanic (85%), White (70%), and married or partnered (65%). Additionally, the sample was highly educated, with three quarters reporting a bachelor’s degree or higher, which may limit the transferability of findings to more racially, ethnically, socioeconomically, and educationally diverse parenting populations.

Future research should therefore include larger and more diverse samples of Deaf parents across communication preferences and modalities, geographic locations, family structures, and cultural identities. Additional work is needed to center the experiences of DeafBlind parents, BIPOC Deaf parents, LGBTQIA+ Deaf parents, single Deaf parents, parents raising CODA children, and Deaf parents of children with disabilities or developmental differences. Longitudinal research would also be valuable for examining how parenting resource needs change across developmental stages, from pregnancy and infancy through adolescence.

## 7. Conclusions

Deaf parents experience widespread communication and information barriers across healthcare, educational, and legal systems that shape their parenting experiences before and after pregnancy. Despite these challenges, supportive family relationships, Deaf community connections, and assistive technologies can facilitate more positive parenting experiences. There is a critical need for increased awareness of the communication and support needs of Deaf parents among healthcare providers, educators, legal professionals, and service systems. Improving access to ASL-based information, ensuring timely accommodations, and enhancing clinician training are essential steps toward promoting equity and supporting optimal parenting outcomes for Deaf families.

## Figures and Tables

**Table 1:** Descriptive Statistics of Demographic Variables (N = 20).

Demographic Variables	N	%
*Gender Identity*		
Female	13	65%
Male	5	25%
Trans man	1	5%
Nonbinary	1	5%
*Ethnicity*		
Non-Hispanic	17	85%
Hispanic	3	15%
*Race*		
White	14	70%
Black/African American	3	15%
Asian	2	10%
Other	1	5%
*Relationship Status*		
Married/Partnered	13	65%
Single	3	15%
Wish not to share	2	10%
Divorced	1	5%
Missed	1	5%
*Highest Education*		
High School	1	5%
Associate	3	15%
Bachelor’s	7	35%
Master’s	6	30%
Doctorate	2	10%
Missed	1	5%

## Data Availability

The data of the present study are unavailable, as participants did not provide their permission to share raw data.

## Abbreviations

The following abbreviations are used in this manuscript:

ASL	American Sign Language
BIPOC	Black, Indigenous, and People of Color
CDI	Certified Deaf Interpreting
CODA	Children of Deaf Adults
IEP	Individualized Education Plan
LGBTQIA+	Lesbian, Gay, Bisexual, Transgender, Queer, Intersex, Asexual, and more
VRI	Video Remote Interpreting

## References

[B1-ejihpe-16-00106] Abedi A., Rostami M., Abedi S., Sudmand N., Movallali G. (2018). Marital satisfaction in deaf couples: A review study. Auditory and Vestibular Research.

[B2-ejihpe-16-00106] Aldalur A., Pick L. H. (2023). Acculturative stress, mental health, and well-being among deaf adults. Journal of Deaf Studies and Deaf Education.

[B3-ejihpe-16-00106] Alhazmi A. A., Kaufmann A. (2022). Phenomenological qualitative methods applied to the analysis of cross-cultural experience in novel educational social contexts. Frontiers in Psychology.

[B4-ejihpe-16-00106] Anderson M. L., Kobek Pezzarossi C. M. (2014). Violence against deaf women: Effect of partner hearing status. Journal of Deaf Studies and Deaf Education.

[B5-ejihpe-16-00106] Anderson M. L., Leigh I. W. (2011). Intimate partner violence against deaf female college students. Violence Against Women.

[B6-ejihpe-16-00106] Ballan M. S., Freyer M. B., Powledge L., Marti C. N. (2017). Intimate partner violence among help-seeking Deaf women: An empirical study. Violence Against Women.

[B7-ejihpe-16-00106] Barnett J., Vasileiou K., Djemil F., Brooks L., Young T. (2011). Understanding innovators’ experiences of barriers and facilitators in implementation and diffusion of healthcare service innovations: A qualitative study. BMC Health Services Research.

[B8-ejihpe-16-00106] Braun V., Clarke V. (2006). Using thematic analysis in psychology. Qualitative Research in Psychology.

[B9-ejihpe-16-00106] Clarke V., Braun V. (2017). Thematic analysis. The Journal of Positive Psychology.

[B10-ejihpe-16-00106] Dotterer A. M. (2022). Diversity and complexity in the theoretical and empirical study of parental involvement during adolescence and emerging adulthood. Educational Psychologist.

[B11-ejihpe-16-00106] Ecklund K., Gamez A. M. (2024). Parenting in the age of intersectionality. Parental influence on educational success and wellbeing.

[B12-ejihpe-16-00106] Fellinger J., Holzinger D., Pollard R. (2012). Mental health of deaf people. The Lancet.

[B13-ejihpe-16-00106] Griese L., Berens E. M., Nowak P., Pelikan J. M., Schaeffer D. (2020). Challenges in navigating the health care system: Development of an instrument measuring navigation health literacy. International Journal of Environmental Research and Public Health.

[B14-ejihpe-16-00106] Hall S., Ballard M., Watson A., Couperus K., Anderson T., Gardner E., Boseman A. (2026). Beyond accommodation: Institutional and policy drivers of healthcare equity for signing deaf adults. BMC Health Services Research.

[B15-ejihpe-16-00106] Helm K. V., Panko T. L., Herschel M., Smith L. D., Mitra M., McKee M. M. (2023). Maternal health experiences of black deaf and hard of hearing women in the United States. Women’s Health Issues.

[B16-ejihpe-16-00106] Holcomb T. K. (2010). Deaf epistemology: The deaf way of knowing. American Annals of the Deaf.

[B17-ejihpe-16-00106] Hoy-Ellis C. P. (2023). Minority stress and mental health: A review of the literature. Journal of Homosexuality.

[B18-ejihpe-16-00106] James T. G., Argenyi M. S., Guardino D. L., McKee M. M., Wilson J. A., Sullivan M. K., Schwartzman E. G., Anderson M. L. (2022). Communication Access in mental health and substance use treatment facilities for deaf American sign language users. Health Affairs.

[B19-ejihpe-16-00106] Kanwal A., Jaleel F., Bashir R., Shahzadi K. (2022). Challenges limiting the role of deaf parents in academics of their children with normal hearing. Sustainable Business and Society in Emerging Economies.

[B20-ejihpe-16-00106] Kuenburg A., Fellinger P., Fellinger J. (2016). Health care access among deaf people. Journal of Deaf Studies and Deaf Education.

[B21-ejihpe-16-00106] Kushalnagar P., Naturale J., Paludneviciene R., Smith S. R., Werfel E., Doolittle R., DeCaro J. (2015). Health websites: Accessibility and usability for American sign language users. Health Communication.

[B22-ejihpe-16-00106] Mauldin L., Leigh I. W., O’Brien C. A. (2020). Lessons learned: How studying cochlear implantation reveals the context in which d/deaf identities are formed. Deaf identities: Exploring new frontiers.

[B23-ejihpe-16-00106] McKee M. M., James T. G., Helm K. V., Marzolf B., Chung D. H., Williams J., Zazove P. (2022). Reframing our health care system for patients with hearing loss. Journal of Speech, Language, and Hearing Research.

[B24-ejihpe-16-00106] McKee M. M., Plegue M., Champlin S., Hill J., Panko T., Buis L. R., Sen A., Paasche-Orlow M. K., Hauser P. C. (2026). Predictors of health literacy among deaf American sign language users. Patient Education and Counseling.

[B25-ejihpe-16-00106] Meyer I. H. (2003). Prejudice, social stress, and mental health in lesbian, gay, and bisexual populations: Conceptual issues and research evidence. Psychological Bulletin.

[B26-ejihpe-16-00106] Mitra S. (2006). The capability approach and disability. Journal of Disability Policy Studies.

[B27-ejihpe-16-00106] National Association of the Deaf (n.d.). Advocacy paper for families seeking accessible PreK-12 education.

[B28-ejihpe-16-00106] Pollard R. Q., Barnett S. (2009). Health-related vocabulary knowledge among deaf adults. Rehabilitation Psychology.

[B29-ejihpe-16-00106] Pollard R. Q., Sutter E., Cerulli C. (2014). Intimate partner violence reported by two samples of deaf adults via a computerized American sign language survey. Journal of Interpersonal Violence.

[B30-ejihpe-16-00106] Powell R. M., Albert S. M. (2021). Barriers and facilitators to compliance with the Americans with disabilities act by the child welfare system: Insights from interviews with disabled parents, child welfare workers, and attorneys. Stanford Law & Policy Review.

[B31-ejihpe-16-00106] Ratakonda S., Panko T. L., Albert S., Smith L. D., Cooley M. M., Mitra M., McKee M. (2025). Wait, what? What’s going on?—Pregnancy experiences of deaf and hard of hearing mothers who do not sign. Birth.

[B32-ejihpe-16-00106] Scotten M. (2015). Parental health literacy and its impact on patient care. Primary Care.

[B33-ejihpe-16-00106] Shakespeare T., Davis L. J. (2010). The social model of disability. The disability studies reader.

[B34-ejihpe-16-00106] Singleton J. L., Tittle M. D. (2000). Deaf parents and their hearing children. Journal of Deaf Studies and Deaf Education.

[B35-ejihpe-16-00106] St. Clair V., Rosenburg P., Mercure E. (2025). Lived experiences of deaf parents: Insights into pride, community, bilingualism, and barriers. Frontiers in Psychology.

[B36-ejihpe-16-00106] Tomaszewski P., Krzysztofiak P., Kowalska J., Hauser P. C. (2025). Internalized oppression and deaf people’s mental health. Scientific Reports.

[B37-ejihpe-16-00106] Trahan K. (2016). An examination of parental experiences in individualized education plans of their deaf children: A qualitative study (Publication No. 10192616). Doctoral Dissertation.

[B38-ejihpe-16-00106] Wilks R. (2025). Deaf legal theory: Challenging the law’s hearing bias. Journal of Deaf Studies and Deaf Education.

[B39-ejihpe-16-00106] Wu C. L., Grant N. C., Leigh I. W., O’Brien C. A. (2020). Intersectionality—Beyond the individual: A look into cultural identity development of Deaf and hard-of-hearing children of multicultural “hearing” families. Deaf identities: Exploring new frontiers.

[B40-ejihpe-16-00106] Zidenberg A. M. (2023). Avoiding the deaf penalty: A review of the experiences of d/deaf individuals in the criminal justice system. Disability & Society.

